# Association of annual percentage changes in anthropometric indices with all-cause mortality: a 14-year longitudinal study of older US adults

**DOI:** 10.3389/fpubh.2026.1894939

**Published:** 2026-07-30

**Authors:** Furong Xu, Jacob E. Earp, Kathleen Woolf, Mary L. Greaney, Bryan J. Blissmer

**Affiliations:** 1 College of Education, University of Rhode Island, Kingston, RI, United States; 2 Department of Kinesiology, University of Connecticut, Storrs, CT, United States; 3 Department of Nutrition and Food Studies, New York University, New York, NY, United States; 4 Department of Public Health, University of Rhode Island, Kingston, RI, United States; 5 Department of Kinesiology, University of Rhode Island, Kingston, RI, United States

**Keywords:** anthropometric index, BMI change, older adults, WC change, weight change

## Abstract

**Introduction:**

Longitudinal anthropometric changes may provide prognostic information beyond single timepoint measurements in older adults. We tested whether the annual percentage change (APC) in five anthropometric metrics (body mass index [BMI], waist circumference [WC], weight-adjusted waist index [WWI], waist-to-height ratio [WHtR], and waist-to-height^0.5 [WHT.5R]) were associated with all-cause mortality in a 14-year U.S. cohort of older adults.

**Methods:**

Using data from the National Health and Aging Trends Study (NHATS) 2011–2024 (*N* = 5,245 after excluding deaths < 2 years), we calculated APC in BMI, WC, WWI, WHtR, WHT.5R using participant specific log-linear models. Survey-weighted Cox proportional hazard models were used to examine APC categories (stable [≤ 2% change] vs. larger gain/loss [> 2% change]) in relation to all-cause mortality, adjusted for baseline age, sex, race, education, annual income, smoking status, homebound status, major chronic disease status, physical frailty, functional status, and cognitive impairment. To ensure the robustness of study findings across all five indices, supplementary restricted cubic spline analyses were performed to validate our primary categorization analysis. Sensitivity analyses included 3- and 5-year lags to minimize reverse causation, explore sex-specific differences, and a two-point APC to confirm findings were not a modeling artifact.

**Results:**

Compared to stable (≤ 2% change) measures, fluctuations in all five anthropometric indices were associated with an elevated mortality risk (pooled HR range: 2.15–9.74; 95% CI boundaries ranged from 1.68 to 17.49; *p* < 0.001). Restricted cubic splines analyses supported a generally V-shaped association between anthropometric changes and mortality (all *p*_non-linearity_ < 0.05). Lagged sensitivity analyses produced findings generally consistent with the primary models (all *p* < 0.05). This elevated risk was observed for both gain and loss categories and were generally consistent across sex-stratified and two-point APC sensitivity analyses.

**Conclusion:**

These findings suggest that stability in anthropometric metrics may reflect a lower-risk physiological profile in older adults. Fluctuations (gain or loss) in the examined anthropometric metrics were associated with higher mortality risk, with generally consistent findings across sensitivity analyses. However, these associations should be interpreted cautiously as anthropometric changes may reflect underlying illness, frailty, or other unmeasured factors.

## 1 Introduction

Anthropometric measures such as body mass index (BMI), waist circumference (WC) are widely used in clinical, community, and research settings because they are low-cost, non-invasive indicators of chronic disease risk (e.g., coronary heart disease, dyslipidemia, type II diabetes, high blood pressure) and mortality (1–3). Five commonly used indices are BMI, WC, weight-adjusted waist index (WWI), waist-to-height ratio (WHtR), and waist-to-height^0.5 (WHT.5R) (1, 3, 4). Despite frequent criticism for failing to discern between lean and fat mass, BMI remains a standard tool in both practice and clinical guidelines (5). However, WC is a better predictor of metabolic risk than BMI because it reflects abdominal adiposity (6). A newer metric, the WWI, measures WC relative to body weight (1, 3). To account for body size differences, WHtR was introduced as a screening tool for central adiposity, while WHT.5R further adjusts this relationship to account for potential height-related bias (7–9).

Previous studies have examined the associations between BMI, WC, and WC-related indices and mortality risk among older adults (4, 10–12). Although the “obesity paradox” has been observed in older populations (12), both increases and decreases in BMI have been associated with higher mortality risk (11–13). Weight change is also recognized as a phenotypic marker of frailty and is a predictor of mortality risk in both middle-aged and older adults (14–17). However, findings regarding WC change and mortality remain inconsistent, with studies reporting null, inverse, or positive associations depending on the population and methodology (18–22).

Most epidemiologic studies rely on single-time-point measurements of body size, which may not capture the dynamic physiological changes that occur with aging. Yet fat distribution can shift with age even at a stable weight (23). These longitudinal changes may reflect underlying processes such as sarcopenia, fat redistribution, or disease progression (24, 25). Focusing on annual percentage change (APC) in anthropometric indices may capture prognostic information and trends that static measures miss. Weight loss and gain in older adults have both been associated with increased mortality risk, though findings remain inconsistent across different populations and measurement approaches (26–28)^.^ In particular, relatively few studies have examined dynamic changes across multiple anthropometric indices simultaneously (26–28).

APC offers a standardized approach for quantifying longitudinal shifts in anthropometric metrics across individuals and follow-up intervals. However, only limited research has examined APC across multiple adiposity indices and the relationship with mortality (26–28), particularly among older US adults. It is not clear whether changes in central adiposity indices offer prognostic value beyond BMI. Furthermore, it is not known if large bidirectional shifts in anthropometry are consistently associated with elevated mortality risk. Addressing this gap is critical because, while BMI remains the primary global standard in clinical guidelines (5), WC provides essential, independent insights into metabolic risk in everyday practice (6).

Therefore, this study examined associations between APC in multiple anthropometric metrics (BMI, WC, WWI, WHtR, and WHT.5R) and all-cause mortality among US adults aged ≥ 65 years. We hypothesized that a greater magnitude of APC, regardless of direction, would be associated with elevated all-cause mortality, and that these indices would demonstrate comparable prognostic performance. These findings may inform clinical surveillance strategies and improve risk stratification in aging populations.

## 2 Methods

### 2.1 Study design

We conducted a secondary analysis of longitudinal data from National Health and Aging Trends Study (NHATS), a nationally representative cohort of Medicare beneficiaries aged ≥ 65 years in the United States (29). The present study used the NHATS 2011 cohort with a follow up period from 2011 to 2024. Among a total of 8,245 adults aged 65 or older at baseline, 3000 were excluded as they did not have at least two data points of self-reported BMI and/or measured WC (*n* = 2,678) or died within the first 2 years of follow-up (*n* = 322). This resulted in 5,245 participants being included in the study sample. This study was approved by the University of Rhode Island Institutional Review Board (IRB # 2410998-2).

### 2.2 Anthropometric indices

Five anthropometric metrics were examined in the present study: BMI, WC, WWI, WHtR, WHT.5R. BMI was calculated by using self-reported height and weight (29). WC was measured following standardized protocol by trained staff. The final annual in-person interview preceded death by an average of 1 to 12 months (30). WWI was calculated using measured WC and self-reported weight [WC (cm)/Weight (kg)^0.5^]. WHtR and WHT.5R were both calculated using WC and height [WHtR = WC (cm)/Height(cm); WHT.5R = WC (cm)/Height (cm)^0.5^] (3). APC for each anthropometric measure was estimated using participant-specific log-linear regression models (31). Each individual model regressed the natural logarithm of the anthropometric measure against time, utilizing all available data points throughout the study period. APC values were categorized as stable (change within 2%; reference), 2% < change ≤ 5, 5% < change ≤ 10, 10% < change. Analyses evaluated both increases and decreases relative to the stable reference group (±2%), which was selected as a pragmatic analytic definition of relative stability around zero annual change. This threshold was not intended to represent a validated clinical cutoff.

### 2.3 All-cause mortality

We utilized NHATS Sensitive Demographic files to identify mortality status (29). These files mainly capture death information through “Last Month of Life” proxy interviews conducted after a participant was confirmed deceased in a subsequent study round (29, 32).

### 2.4 Confounding variables

Baseline characteristics were adjusted across three domains: socio-demographics, lifestyle, and clinical functional status. Participant demographic characteristics were categorized as follows: age (65–74, 75–84, or 85 + years), race/ethnicity (White, Black, Hispanic, or Other), educational attainment (high school graduate or less, or college graduate or more), annual income (29). Lifestyle characteristics include smoking status (non-smokers or ever smokers) and homebound status (homebound, semi-homebound, not homebound) (29). For smoking status classification, respondents who currently smoke regularly or have smoked 100 or more cigarettes in their lifetime were classified as ever smokers (33). The lifetime total determined by multiplying the years of regular smoking by the average cigarettes smoked per day, which was obtained from five smoking-related questions from NHATS (29, 33).

Additionally, baseline clinical confounders included major chronic diseases, physical frailty, functional status, and cognitive impairment, which were captured via structured, annual in-person interviews (29, 34). Specifically, major chronic disease status was determined using available baseline indicators for three major chronic diseases, including heart disease, diabetes, and cancer, with participants classified as having none or one or more of these conditions (29, 35). Physical frailty was evaluated based on the five established criteria of the Fried physical frailty phenotype as operationalized within NHATS, utilizing a combination of self-reported interview questions (exhaustion, low physical activity, weight loss) and objective physical performance assessment (gait speed, grip strength testing), with a score of three or more indicating frailty (14, 36). Functional status was assessed by checking how well individuals handle everyday tasks, grouping them as either more able or less able (37). Finally, cognitive status was evaluated using a three-part protocol tracking self- or proxy-disclosed diagnoses, an 8-item screening instrument, and objective cognitive test results. Participants were categorized as having dementia based on a reported medical diagnosis or positive screening with objective deficits in one or more cognitive domains (35). Conversely, all other participants were classified as having no dementia (35).

### 2.5 Statistical analysis

Baseline analytic weights were applied in all analyses to ensure population representativeness (38). Data are presented as weighted means ± standard errors for continuous variables and n (weighted percentage) for categorical variables. The primary outcome was all-cause mortality, determined through NHATS-linked mortality data, with follow-up from 2011 through 2024. Person-time was calculated from baseline until death or censoring at the end of follow up. Survey-weighted Cox proportional hazards models were used (PROC SURVEYPHREG, SAS 9.4) to examine association between the APC in anthropometric indices and all-cause mortality risk, adjusted for baseline age, sex, race/ethnicity, education, annual income, smoking status, homebound status, major chronic diseases, physical frailty, functional status, and cognitive impairment. The proportional hazards assumption was verified using the Schoenfeld residuals test for time-varying BMI and WC categories (*p* > 0.05 for all). Model discrimination was assessed via Harrell C-statistics, with values > 0.7 indicating good performance. To complement the categorical models, we performed restricted spline cubic analyses across all five anthropometric metrics to evaluate the continuous functional form of these associations. Spline figures for all five anthropometric measures are included alongside the primary figures in the main manuscript. To examine the potential influence of early deaths and reverse causation, sensitivity analysis was performed excluding participants who died during the first 3 and 5 years of follow-up (39, 40). Additional sensitivity analyses included a sex-specific evaluation, and a two-point (first vs. last) APC analysis confirmed that the anthropometric-mortality association was not a modeling artifact. Exploratory analyses assessed the relationship between the APC of WC and BMI changes via the concordance correlation coefficient. All analyses were conducted using SAS 9.4.

## 3 Results

Among 5,245 adults analyzed in the present study, 55.5% were females, 16.8% were racial/ethnic minorities, 18.8% had a high school education or less, 6.7% were homebound or semi-homebound, and 53.1% were ever smokers. At baseline, 10.2% of participants were 85 years or older (Table 1). The number of participants and outcome events in each APC category are reported in Table 2.

**Table 1:** Characteristics of study participants at baseline.

Baseline Characteristics	Total	Male	Female	*p* value
	*N* = 5,245	*n* = 2,244 (44.5%)	*n* = 3,001 (55.5%)	
Age (yrs), *n* (weighted %)				
65–74	2,269 (56.7)	1,047 (59.7)	1,222 (54.2)	<0.001*
75–84	2,115 (33.1)	885 (32.1)	1,230 (32.1)	0.21
85+	861 (10.2)	312 (8.1)	549 (11.8)	<0.001*
Race, *n* (weighted %)				
White	3,713 (83.2)	1,600 (83.1)	2,113 (83.3)	0.841
Black	1,092 (7.7)	438 (7.1)	654 (8.3)	0.016*
Hispanic	280 (6.1)	129 (6.4)	151 (5.8)	0.474
Other	129 (3.0)	67 (3.5)	62 (2.6)	0.13
Education, *n* (weighted %)				
High school graduate or less	1,236 (18.8)	531 (19.1)	705 (18.6)	0.617
College or above	3,983 (81.2)	1705 (80.9)	2,278 (81.4)	0.617
Annual income, *n* (weighted %)				
<$27,600	2,444 (39.6)	796 (30.3)	1,648 (47.1)	<0.001*
$27,600–$41,999	925 (17.6)	437 (18.0)	488 (17.3)	0.498
$42,000–$63,999	788 (16.4)	389 (17.7)	399 (15.4)	0.088
$64,000–$107,999	686 (16.3)	376 (20.0)	310 (13.4)	<0.001*
≥$108,000	402 (10.0)	246 (14.0)	156 (6.7)	<0.001*
Homebound status				
Homebound	192 (3.0)	39 (1.5)	153 (4.1)	<0.001*
Semi-homebound	250 (3.7)	51 (1.8)	199 (5.2)	<0.001*
Not homebound	4,803 (93.3)	2,154 (96.6)	2,649 (90.7)	<0.001*
Ever smokers	2,701 (53.1)	1,469 (65.0)	1,232 (43.5)	<0.001*
Major chronic diseases	2,747 (50.6)	1,263 (54.2)	1,484 (47.8)	<0.001*
Physical frailty	1,577 (50.5)	536 (39.0)	1,041 (59.9)	<0.001*
Functional status	4,899 (94.7)	2029 (92.0)	2,870 (96.9)	<0.001*
Cognitive impairment	956 (14.2)	440 (15.7)	516 (13.1)	0.009*
BMI (kg/m^2^)	27.8 ± 0.1	27.9 ± 0.1	27.7 ± 0.1	0.286
WC (cm)	100.2 ± 0.5	104.7 ± 0.3	96.6 ± 0.8	<0.001*
WWI	11.4 ± 0.1	11.3 ± 0.0	11.4 ± 0.1	<0.001*
WHtR	0.6 ± 0.0	0.6 ± 0.0	0.6 ± 0.0	<0.001*
WHT.5R	7.7 ± 0.0	7.9 ± 0.0	7.6 ± 0.1	<0.001*

Data are presented as weighted means ± standard errors (SE) for continues variables whereas *n* (weighted %) for categorical variables. BMI, body mass index; WC, waist circumference; WWI, weight-adjusted waist index; WHtR, Waist-to-height ratio; and WHT.5R, waist-to-height^0.5 *p < 0.05.

**Table 2:** The APC change in anthropometric indices and their association with all-cause mortality.

Anthropometric metric	Event/No. at risk	Adjusted HR (95% CI)	*p* value	Harrell C statistic (95% CI)
BMI change				
Stable (within 2% change)	1,185/3,513	1.00 (REF)	—	0.746 (0.733, 0.759)
5% ≥ Decreased >2%	450/874	2.15 (1.80, 2.58)	<0.001*	
10% ≥ Decreased >5%	154/276	5.13 (4.08, 6.47)	<0.001*	
Decreased >10%	51/91	7.13 (4.43, 11.47)	<0.001*	
5% ≥ Increased >2%	132/321	2.92 (2.14, 3.98)	<0.001*	
10% ≥ Increased >5%	45/117	3.01 (1.68, 5.39)	<0.001*	
Increased >10%	24/53	9.74 (5.43, 17.49)	<0.001*	
WC change				
Stable (within 2% change)	1,382/3,690	1.00 (REF)	—	0.754 (0.741, 0.767)
5% ≥ Decreased >2%	298/599	2.72 (2.25, 3.27)	<0.001*	
10% ≥ Decreased >5%	73/188	6.65 (4.67, 9.47)	<0.001*	
Decreased >10%	29/79	7.44 (3.57, 15.51)	<0.001*	
5% ≥ Increased >2%	181/465	3.10 (2.50, 3.85)	<0.001*	
10% ≥ Increased >5%	62/164	7.05 (4.77, 10.42)	<0.001*	
Increased >10%	16/60	5.79 (2.66, 12.63)	<0.001*	
WWI change				
Stable (within 2% change)	1,486/3,922	1.00 (REF)	—	0.755 (0.743, 0.768)
5% ≥ Decreased >2%	170/378	3.94 (3.20, 4.87)	<0.001*	
10% ≥ Decreased >5%	44/134	7.69 (4.97, 11.91)	<0.001*	
Decreased >10%	25/66	4.70 (2.79, 7.91)	<0.001*	
5% ≥ Increased >2%	217/475	3.39 (2.74, 4.20)	<0.001*	
10% ≥ Increased >5%	70/181	4.05 (2.94, 5.58)	<0.001*	
Increased >10%	29/89	4.85 (2.77, 8.47)	<0.001*	
WHtR change				
Stable (within 2% change)	1,359/3,668	1.00 (REF)	—	0.750 (0.737, 0.763)
5% ≥ Decreased >2%	271/552	2.56 (2.11, 3.10)	<0.001*	
10% ≥ Decreased >5%	77/188	5.28 (3.34, 8.36)	<0.001*	
Decreased >10%	36/87	5.85 (3.41, 10.04)	<0.001*	
5% ≥ Increased >2%	206/493	2.68 (2.21, 3.25)	<0.001*	
10% ≥ Increased >5%	63/179	6.53 (5.01, 8.51)	<0.001*	
Increased >10%	29/78	4.60 (2.53, 8.35)	<0.001*	
WHT.5R change				
Stable (within 2% change)	1,370/3,690	1.00 (REF)	—	0.750 (0.737, 0.763)
5% ≥ Decreased >2%	278/568	2.52 (2.09, 3.04)	<0.001*	
10% ≥ Decreased >5%	81/190	5.24 (3.39, 8.12)	<0.001*	
Decreased >10%	34/84	5.86 (3.31, 10.36)	<0.001*	
5% ≥ Increased >2%	185/463	2.61 (2.15, 3.17)	<0.001*	
10% ≥ Increased >5%	67/177	6.76 (5.17, 8.83)	<0.001*	
Increased >10%	26/73	3.76 (2.11, 6.70)	<0.001*	

APC (annual percentage change) was estimated to use Log-Linear Model for every participant. The associations between anthropometric indices and all-cause mortality were examined via multiple analysis, the models adjusted for baseline age, sex, race, education, annual income, ever smokers, homebound status, major chronic disease status, physical frailty, functional status, and cognitive impairment. HR, hazard ratio, CI, confidential interval, BMI, body mass index, WC, waist circumference, WWI, weight-adjusted waist index, WHtR, Waist-to-height ratio, and WHT.5R, waist-to-height^0.5; *p < 0.05.

**Figure 1:**
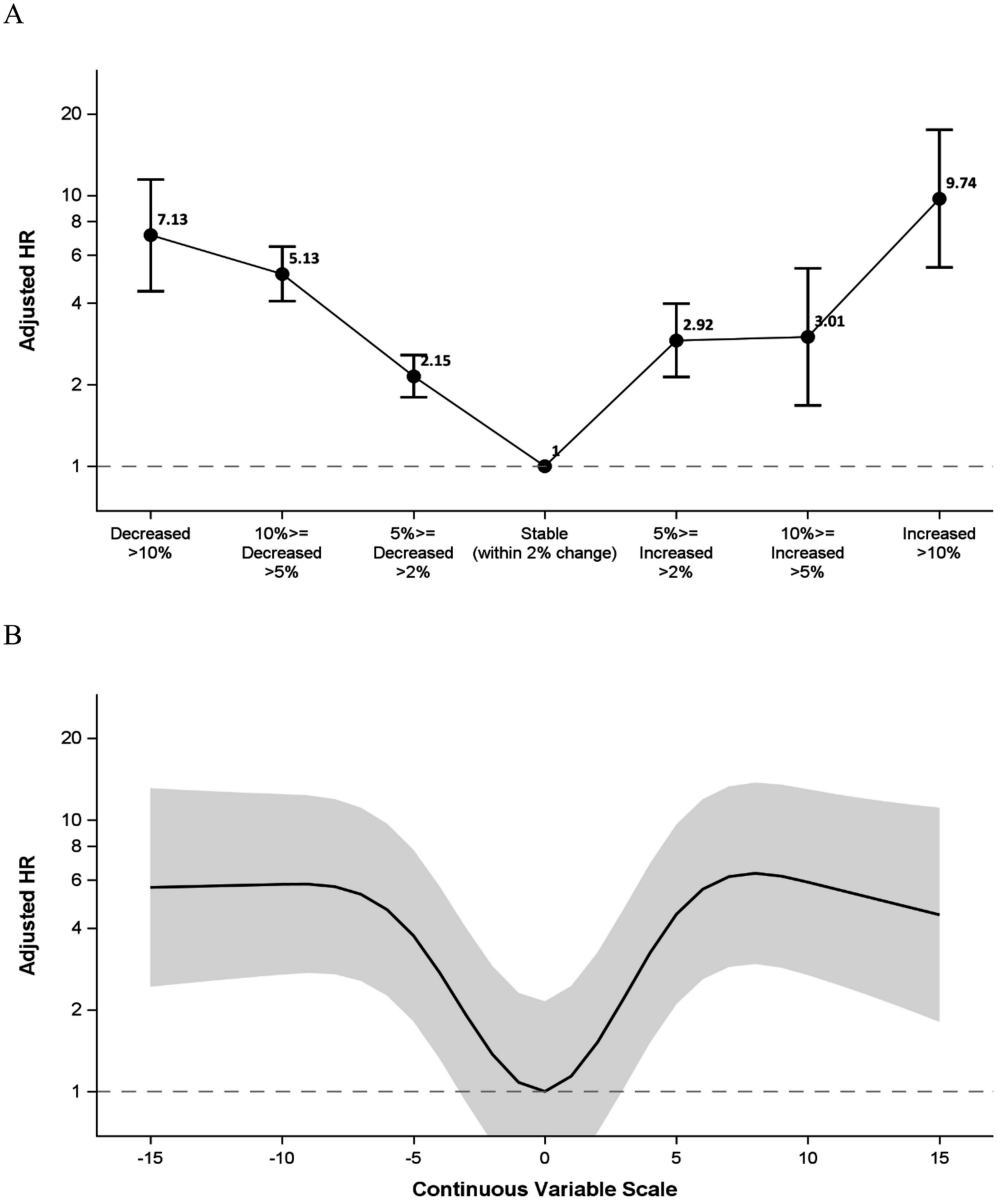
Association between BMI APC and all-cause mortality risk. **(A)** Multivariable-adjusted HR across discrete categorical BMI APC intervals (primary analysis). **(B)** Association of BMI APC with HR for mortality using a restricted cubic spline model with 5 knots (supplementary analysis). Reference lines denote the baseline hazard (HR = 1.0). BMI, body mass index; APC, annual percentage change; HR, hazard ratios.

**Figure 2:**
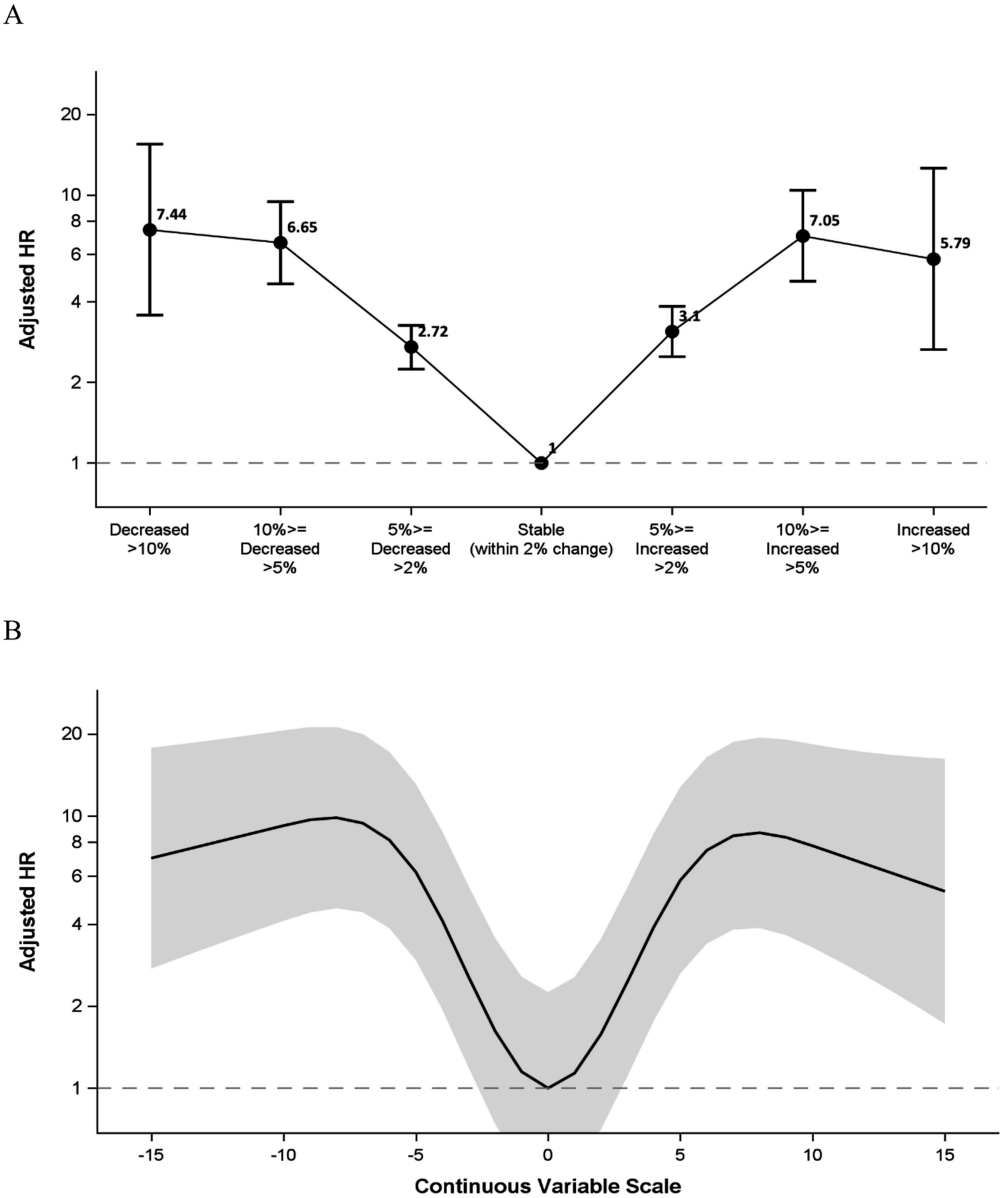
Association between WC APC and all-cause mortality risk. **(A)** Multivariable-adjusted HR across discrete categorical WC APC intervals (primary analysis). **(B)** Association of WC APC with HR for mortality using a restricted cubic spline model with 5 knots (supplementary analysis). Reference lines denote the baseline hazard (HR = 1.0). WC, waist circumference; APC, annual percentage change; HR, hazard ratios.

**Figure 3:**
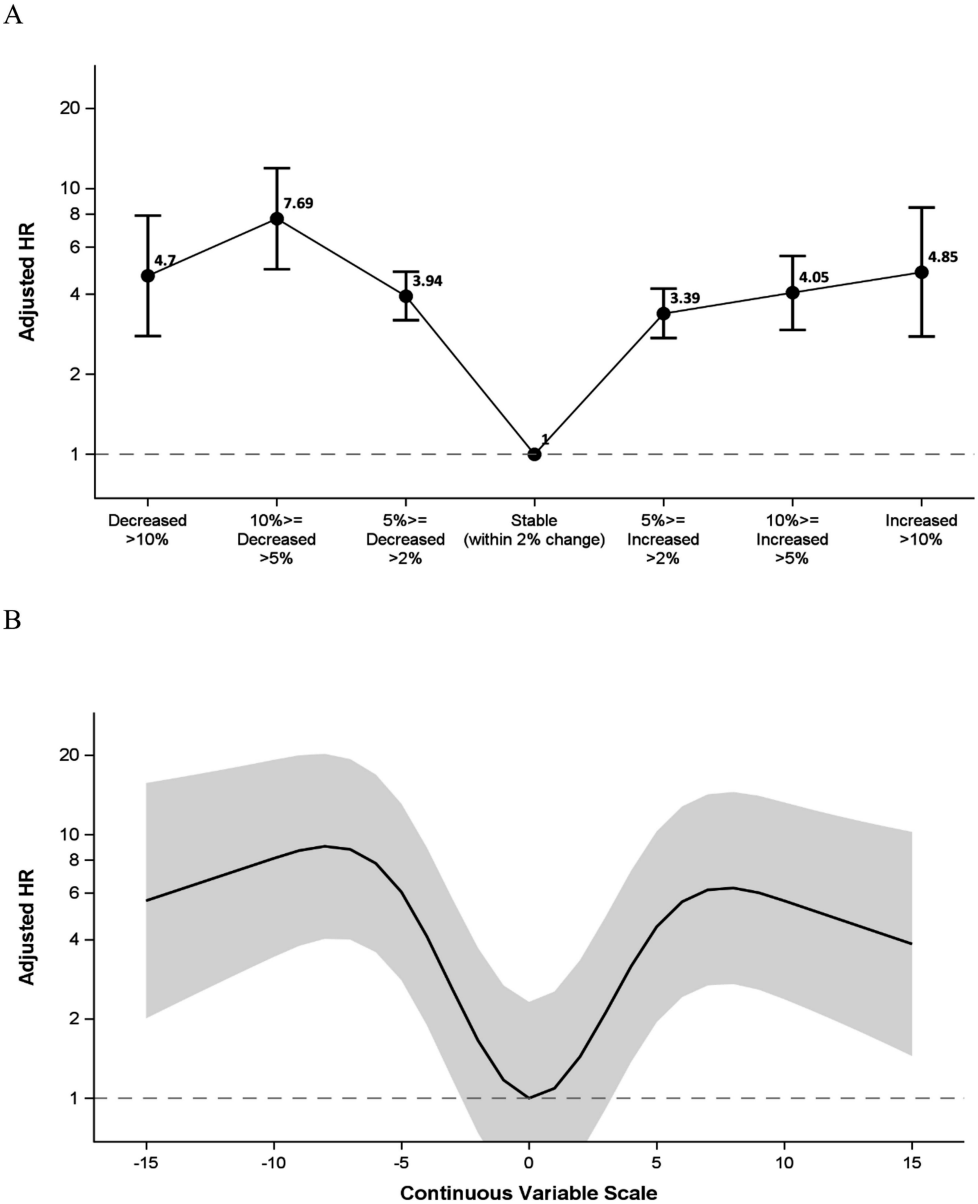
Association between WWI APC and all-cause mortality risk. **(A)** Multivariable-adjusted HR across discrete categorical WWI APC intervals (primary analysis). **(B)** Association of WWI APC with HR for mortality using a restricted cubic spline model with 5 knots (supplementary analysis). Reference lines denote the baseline hazard (HR = 1.0). WWI, weight-adjusted waist index; APC, annual percentage change; HR, hazard ratios.

**Figure 4:**
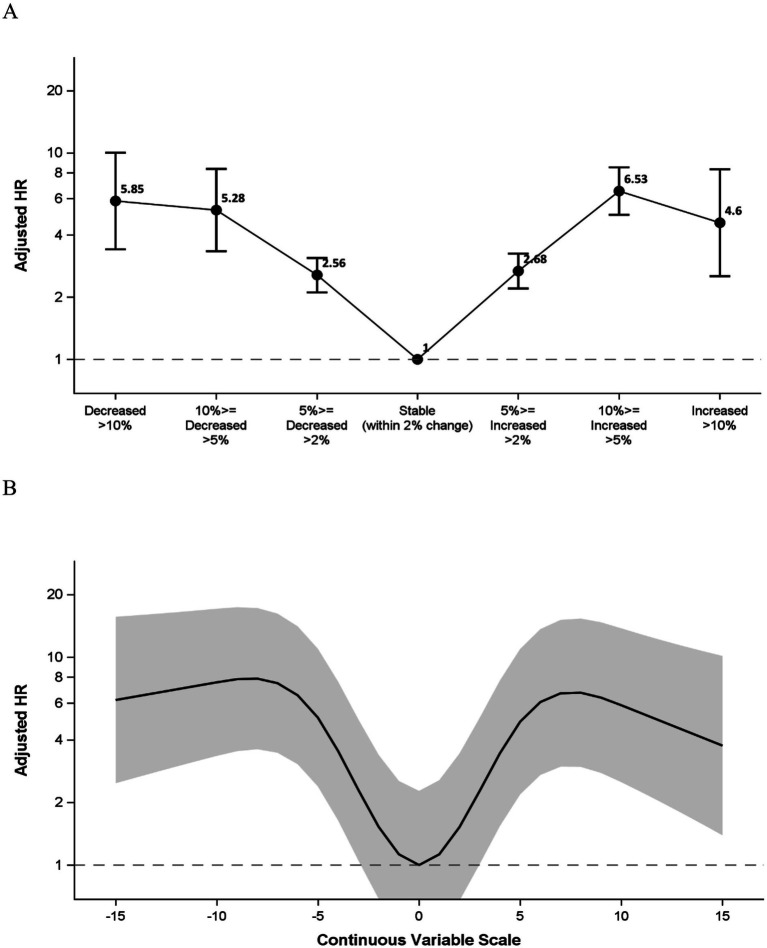
Association between WHtR APC and all-cause mortality risk. **(A)** Multivariable-adjusted HR across discrete categorical WHtR APC intervals (primary analysis). **(B)**. Association of WHtR APC with HR for mortality using a restricted cubic spline model with 5 knots (supplementary analysis). Reference lines denote the baseline hazard (HR = 1.0). WHtR, waist-to-height ratio; APC, annual percentage change; HR, hazard ratios.

**Figure 5:**
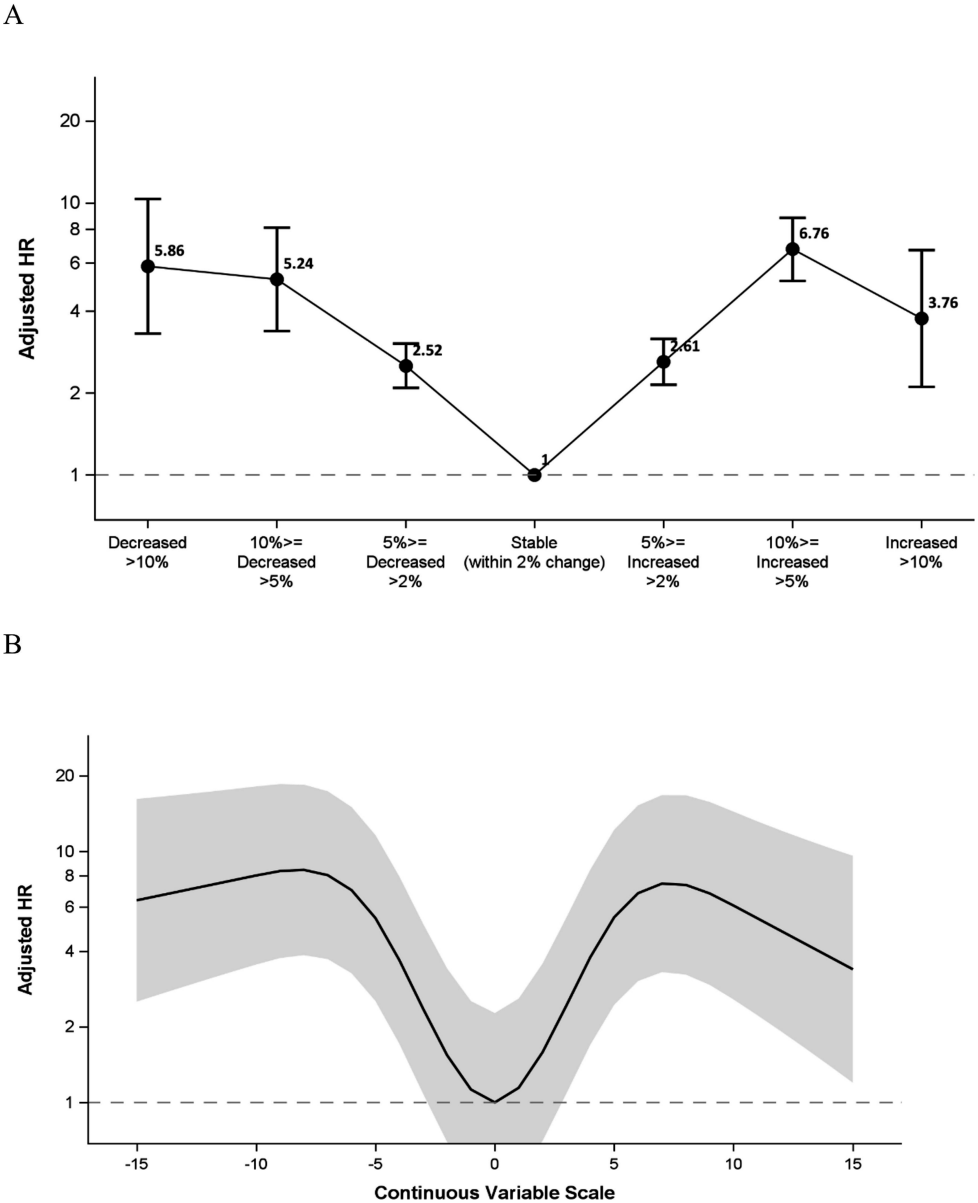
Association between WHT.5R APC and all-cause mortality risk. **(A)** Multivariable-adjusted HR across discrete categorical WHT.5R APC intervals (primary analysis). **(B)** Association of WHT.5R APC with HR for mortality using a restricted cubic spline model with 5 knots (supplementary analysis). Reference lines denote the baseline hazard (HR = 1.0). WHT.5R, waist-to-height^0.5; APC, annual percentage change; HR, hazard ratios.

Association between APC in BMI, WC, WWI, WHtR, WHT.5R, and all-cause mortality showed a generally graded pattern. Greater magnitude of change, whether increase or decrease, was associated with progressively higher mortality risk compared with stable values (≤ 2% change) (Table 2; Figures 1–5). Compared with the reference group, greater mortality risk was observed among individuals with > 10% BMI APC changes (increased: HR 9.74, 95% [5.43, 17.49], decreased: HR 7.13, 95% CI [4.43, 11.47]) than individuals with changes between 5 to 10% (increased: HR 3.01, 95% [1.68, 5.39], decreased: HR 5.13, 95% CI [4.08, 6.47]), and followed by individual with annual percentage change between 2% and 5% (increased: HR 2.92, 95% [2.14, 3.98], decreased: HR 2.15, 95% CI [1.80, 2.58]) (see Table 2; Figure 1). A similar pattern was observed in WC, WWI, WHtR and WHT.5R changes (Figures 2–5). Detailed estimates are presented in Table 2.

The Harrell C statistics indicated that the predictive power of all anthropometric measures were comparable (see Table 2): [BMI, C-statistic 0.746 (95% CI 0.733–0.759); WC, C-statistic 0.754 (95% CI 0.741–0.767); WWI, C-statistic 0.755 (95% CI 0.743–0.768); WHtR C-statistic 0.750 (95% CI 0.737–0.763); WHT.5R, C-statistic 0.750 (95% CI 0.737–0.763)]. Moreover, the exploratory analysis showed that the relationship between BMI and WC was weak (CCC = 0.21 [95% CI: 0.18, 0.23]) (Supplementary Figures S1, S2).

Additionally, the findings of the sensitivity analyses examining the association between anthropometric metrics and mortality, excluding participants who passed away within the first 3 (*n* = 529 excluded) and 5 years of follow-up (*n* = 960 excluded) remained consistent with the primary findings, supporting the robustness of the findings (Supplementary Table S1). Sensitivity analyses also revealed the consistency between anthropometric metrics and mortality across both sexes (Supplementary Table S2). Furthermore, results from the two-point APC analysis mirrored the primary findings, suggesting that the findings were not dependent on whether APC was estimated using the log-linear approach or the two-point approach (Supplementary Table S3).

## 4 Discussion

Study findings add to the evidence on body-composition dynamics by examining the associations between APC in five common anthropometric indices and all-cause mortality risk among US adults 65 years or older over a 14-year follow-up. This study provides longitudinal evidence into how the magnitude of change in these indices predicts long-term mortality relative to stable reference groups. The predictive performance of each index for all-cause mortality was also evaluated to bridge the gap between statistical observation and practical application in clinical settings. We observed that larger bidirectional changes in BMI, WC, WWI, WHtR, and WHT.5R were consistently associated with increased mortality risk over 14 years. These associations were graded, with a greater magnitude of change corresponding to a higher risk, which was similar across all indices. Predictive discrimination was comparable across measures, and concordance between BMI and WC changes was limited, suggesting that different indices capture distinct aspects of physiological change. These findings suggest that relative stability in anthropometric metrics may reflect a lower-risk physiological profile in older adults. Rather than indicating that stability itself is protective, these results are likely to reflect that substantial changes, whether increases or decreases, serve as markers of underlying physiological dysregulations.

The findings indicated that BMI and WC APCs were associated with increased mortality risk, which is broadly consistent with existing research (11, 12, 15–17, 27, 28). Despite the “obesity paradox” noted in older populations, studies have shown that both increases and decreases in BMI are associated with elevated mortality risk (11, 12). This apparent paradox may reflect competing risks, where higher BMI is protective in certain clinical contexts, while dynamic changes in BMI signal physiological instability. A decrease in BMI in older adults is often associated with loss of lean mass, reduced bone density, and sarcopenia, all of which contribute to increased frailty and mortality risk (41–43). Conversely, increases in BMI may reflect excess adiposity, contributing to metabolic dysfunction, cardiovascular strain, and chronic disease development (44). Taken together, these findings suggest that relative stability in BMI may reflect a lower-risk physiological profile, whereas substantial deviations, regardless of direction, may indicate underlying health deterioration.

On the other hand, APC in WC demonstrated similar associations with mortality risk, which is supported by some of the previous studies but not others (18–22). However, direct comparisons are challenging due to methodological differences in measurement of WC and differing age, and race/ethnicity of study participants (18–22). The present study extends this literature by examining WC changes over a longer follow-up period (14 years) in a nationally representative sample of older U.S. adults. This longer follow-up may capture cumulative physiological changes that shorter studies fail to detect. In this context, decreases in WC may reflect underlying disease processes, such as cancer or depression (45, 46); whereas increases may indicate an accumulation of visceral adiposity and associated metabolic risk (47).

The present study additionally examined APC in WWI, WHtR, WHT.5R, which are innovative indices designed to better capture central adiposity. WWI adjusts WC for body weight, whereas WHtR and WHT.5R account for body size and height-related bias (4). Although research on longitudinal changes in these indices remains limited, one prior study reported that both increases and decreases in WHtR over a 3-year period were associated with higher mortality risk compared to stable values in a younger Asian population (48). The present study builds on this evidence by demonstrating similar bidirectional associations across multiple central adiposity indices in an older U.S. population over a substantially longer follow-up period. Our findings revealed that the greater magnitude of change in these metrics, whether a gain or loss, is consistently linked to an increased risk of mortality. However, mortality risk was not strictly linear for some metrics (e.g., WWI, WHtR and WHT.5R) across sex-specific subgroups. These non-linear patterns may reflect heterogeneity in underlying health status, competing risks, or differential survival among subgroups rather than a simple dose–response relationship. For example, individuals with extreme anthropometric changes may represent clinically distinct populations with varying disease burdens or treatment histories.

The findings of the current study are broadly consistent with prior longitudinal studies showing that both weight gain and weight loss in later life are associated with elevated mortality risk (27). Our results extend this literature by examining APC across multiple anthropometric indices simultaneously and by demonstrating comparable predictive performance across measures. The weak concordance between BMI and WC change indicated that although both BMI and WC change affect health, they reflect different physiological changes. BMI reflects overall body mass changes, whereas WC reflects central fat distributions and visceral adiposity (49).

The present study also compared the predictive performance of the anthropometric indices for all-cause mortality risk among older US adults. The study found that all examined anthropometric indices performed about the same, differences in Harrell C-statistics across indices were small, although WWI and WC change were slightly more accurate than the others. No substantial sex differences were observed. As WWI, WHtR, WHT.5R are derived from WC, these findings suggest that WC-related changes may serve as a common underlying marker of central adiposity. At the same time, BMI and WC appear to function as complementary, rather than redundant, indicators of health risk (50). Measuring both BMI and WC provides a more comprehensive health risk assessment than relying on either one alone. Moreover, while the predictive power is consistent across examined measures, establishing index-specific thresholds facilitates more precise risk stratification for older adults. Consequently, further study is needed to determine the optimal cut point for APC in BMI and WC, which could improve mortality predictability and provide a standardized framework for comparing the diagnostic accuracy of these indices in older adults.

Several biological mechanisms may explain the bidirectional associations observed in the current study. Increases in anthropometric metrics likely reflect progressive accumulation of adiposity, which contributes to cardiometabolic dysfunction through insulin resistance, inflammation, and vascular changes (51). Conversely, decreases in anthropometry in older adults may reflect underlying illness, sarcopenia, or frailty-related weight loss, all of which are associated with increased mortality risk (24, 25). These competing mechanisms highlight that anthropometric change is not inherently harmful but rather may serve as a marker of underlying physiological processes.

### 4.1 Strengths and limitations

The study design (longitudinal) and duration (14 years) are the strengths of the present study. Furthermore, the data used (NHATS) is a nationally representative study of Medicare beneficiaries aged 65 or older in the United States (29). Moreover, the five anthropometric indices are widely used in the existing literature (1, 3, 4), but the associations of their APC and all-cause mortality in older adults are not well established. Longitudinal assessment of these anthropometric markers offers insight into physiological decline that static measurements overlook. Our categorical findings were further supported by the restricted cubic spline analyses. By mapping the continuous relationship between anthropometric APC and mortality, the spline models supported a generally V-shaped pattern and suggested that the categorical approach did not obscure non-linear risk patterns. Furthermore, the validity of our findings is reinforced by the consistency observed in our sensitivity analyses. The 3- and 5-year lag analyses reduced concern that the findings were driven primarily by deaths occurring soon after baseline, although they cannot eliminate reverse causation from preclinical diseases. Also, using the sensitivity two-point anthropometric APC analysis alongside primary log-linear approach strengthens our findings by demonstrating that anthropometric APC-mortality association is robust to the trend estimation method. This alignment confirms that our conclusions are mathematically stable and not an artifact of complex statistical modeling. In the context of aging, the preservation of weight stability might be a superior predictor of health outcomes compared to absolute value, as it accounts for the complex interplay of muscle loss and fat accumulation (52).

The present study has several limitations that should be considered when interpreting the results. First, we did not distinguish between intentional and unintentional weight loss. Consequently, observed BMI changes may not accurately reflect the causal effect of weight management and may potentially conflate health-seeking behaviors with illness-related decline. Secondly, performing sex-specific subgroup analyses, especially when adjusting for several confounding variables, can reduce statistical power and increase the risk of unstable estimates and model overfitting. While we collapsed sparse categories to minimize unstable estimates, model overfitting in subgroup analysis cannot be entirely ruled out. These exploratory sex-specific subgroup findings should therefore be interpreted with caution. Third, potential reverse causality should be considered, as undiagnosed conditions or early-stage conditions like dementia often manifest with cachexia, causing a drop in BMI that reflects disease progression toward mortality (53), rather than an independent risk factor. The primary analytic sample excluded participants who died within the first 2 years of follow-up, and additional 3- and 5-year exclusion analyses were conducted to assess the potential influence of early deaths. Finally, residual confounding is an inherent limitation in observational studies of older adults. We reduced this concern by adjusting for baseline major chronic disease status, physical frailty, functional status, or cognitive impairment. Nonetheless, certain unmeasured factors such as severe subclinical disease or nutritional status may still contribute to residual confounding. Therefore, our conclusions should be interpreted with caution until validated in populations with more comprehensive clinical data.

## 5 Conclusion

The present study found that changes in all five anthropometric indices were associated with increased mortality risk. These results highlight the clinical relevance of longitudinal stability in body size and adiposity, whether measured by overall body mass (BMI) or fat distribution (WC and derived indices). All indices demonstrated comparable predictive performance. The findings suggest that clinicians may consider monitoring longitudinal changes in body size as indicators of physiological vulnerability, rather than relying solely on static thresholds. However, these measures should be interpreted with caution as they do not distinguish between muscle mass and fat loss, nor between intentional weight loss or illness-induced wasting. They should be viewed as preliminary signals that require deeper clinical context, particularly in aging populations where weight stability is often the primary driver of longevity.

## Data Availability

Publicly available datasets were analyzed in this study. This data can be found here: the National Health & Aging Trends Study (NHATS). Public use files are accessible to researchers upon registration through the NHATS website: https://www.nhats.org/data-access.
