# Supplementary material for: Association of annual percentage changes in anthropometric indices with all-cause mortality: a 14-year longitudinal study of older US adults
Source: Front Public Health. 2026 Jul 30;14:1894939. doi: 10.3389/fpubh.2026.1894939 (PMC13471147; doi:10.3389/fpubh.2026.1894939)
Supplement: Supplementary file 1 [file Data_Sheet_1.pdf]

Supplementary Table S1. Association between annual changes in five anthropometric metrics and all-cause mortality across multiple lag structures.

Anthropometric Metrics	3-year lag analysis (N= 5038)			5-year lag analysis (N=4607)		
	**Event / No. at risk**	**Adjusted HR (95% CI)**	***P* value**	**Event / No. at risk**	**Adjusted HR (95% CI)**	***P* value**
**BMI change**						
Stable (within 2% change)	1124 / 3452	1.00 (Ref)	-	932 / 3260	1.00 (Ref)	-
5% ≥ Decreased >2%	405 / 829	2.16 (1.79, 2.62)	<0.001*	292 / 716	2.06 (1.65, 2.57)	<0.001*
10% ≥ Decreased >5%	128 / 250	5.45 (4.26, 6.99)	<0.001*	66 / 188	6.65 (4.70, 9.41)	<0.001*
Decreased >10%	22 / 62	4.01 (2.34, 6.88)	<0.001*	11 / 51	3.62 (1.76, 7.45)	<0.001*
5% ≥ Increased >2%	107 / 296	2.63 (1.88, 3.67)	<0.001*	77 / 266	2.97 (1.89, 4.67)	<0.001*
10% ≥ Increased >5%	33 / 105	2.41 (1.29, 4.50)	0.007*	20 / 92	2.16 (1.18, 3.96)	0.013*
Increased >10%	15 / 44	11.41 (5.61, 23.22)	<0.001*	5 / 34	7.44 (1.75, 31.55)	0.007*
**WC change**						
Stable (within 2% change)	1313 / 3621	1.00 (Ref)	-	932 / 3260	1.00 (Ref)	-
5% ≥ Decreased >2%	256 / 557	2.68 (2.17, 3.30)	<0.001*	167 / 468	2.58 (1.97, 3.38)	<0.001*
10% ≥ Decreased >5%	49 / 164	6.00 (4.11, 8.74)	<0.001*	16 / 131	6.59 (3.64, 11.93)	<0.001*
Decreased >10%	12 / 62	3.88 (1.96, 7.66)	<0.001*	6 / 56	5.40 (2.00, 14.58)	0.001*
5% ≥ Increased >2%	152 / 436	3.14 (2.43, 4.05)	<0.001*	97 / 381	3.12 (2.33, 4.19)	<0.001*
10% ≥ Increased >5%	43 / 145	8.68 (5.19, 14.52)	<0.001*	12 / 114	6.01 (2.38, 15.22)	<0.001*
Increased >10%	9 / 53	4.95 (1.96, 12.55)	0.001*	3 / 47	3.18 (1.75, 5.79)	<0.001*
**WWI change**						
Stable (within 2% change)	1416 / 3852	1.00 (Ref)	-	932 / 3260	1.00 (Ref)	-
5% ≥ Decreased >2%	128 / 336	3.39 (2.64, 4.34)	<0.001*	64 / 272	2.94 (2.06, 4.18)	<0.001*

10% ≥ Decreased >5%	29 / 119	5.26 (3.42, 8.08)	<0.001*	14 / 104	5.24 (2.58, 10.64)	<0.001*
Decreased >10%	14 / 55	3.30 (1.98, 5.51)	<0.001*	8 / 49	4.76 (2.38, 9.51)	<0.001*
5% ≥ Increased >2%	176 / 434	3.18 (2.52, 4.03)	<0.001*	105 / 363	2.76 (2.06, 3.69)	<0.001*
10% ≥ Increased >5%	52 / 163	4.40 (2.96, 6.53)	<0.001*	21 / 132	2.98 (1.79, 4.95)	<0.001*
Increased >10%	19 / 79	3.97 (1.98, 7.94)	<0.001*	9 / 69	2.22 (0.91, 5.41)	0.079
**WHtR change**						
Stable (within 2% change)	1292 / 3601	1.00 (Ref)	-	932 / 3260	1.00 (Ref)	-
5% ≥ Decreased >2%	231 / 512	2.48 (2.03, 3.03)	<0.001*	146 / 427	2.36 (1.83, 3.04)	<0.001*
10% ≥ Decreased >5%	53 / 164	4.24 (2.69, 6.70)	<0.001*	24 / 135	3.91 (2.17, 7.03)	<0.001*
Decreased >10%	17 / 68	3.86 (2.44, 6.12)	<0.001*	9 / 60	4.76 (2.75, 8.22)	<0.001*
5% ≥ Increased >2%	177 / 464	2.66 (2.15, 3.28)	<0.001*	115 / 402	2.47 (1.85, 3.30)	<0.001*
10% ≥ Increased >5%	42 / 158	7.23 (5.01, 10.42)	<0.001*	14 / 130	6.60 (2.64, 16.49)	<0.001*
Increased >10%	22 / 71	4.53 (2.24, 9.18)	<0.001*	11 / 60	2.68 (1.39, 5.20)	0.004*
**WHT.5R change**						
Stable (within 2% change)	1300 / 3620	1.00 (Ref)	-	932 / 3260	1.00 (Ref)	-
5% ≥ Decreased >2%	239 / 529	2.47 (2.02, 3.01)	<0.001*	149 / 439	2.34 (1.81, 3.02)	<0.001*
10% ≥ Decreased >5%	57 / 166	4.56 (2.91, 7.15)	<0.001*	24 / 133	4.31 (2.44, 7.62)	<0.001*
Decreased >10%	15 / 65	3.44 (2.18, 5.43)	<0.001*	9 / 59	4.69 (2.71, 8.13)	<0.001*
5% ≥ Increased >2%	157 / 435	2.57 (2.08, 3.17)	<0.001*	105 / 383	2.41 (1.79, 3.24)	<0.001*
10% ≥ Increased >5%	47 / 157	7.80 (5.48, 11.10)	<0.001*	14 / 124	6.36 (2.41, 16.80)	<0.001*
Increased >10%	19 / 66	3.54 (1.82, 6.88)	<0.001*	11 / 58	2.85 (1.59, 5.11)	<0.001*

Note: the sex specific associations between anthropometric changes and all-cause mortality examined via multiple analysis and models adjusted for baseline age, race, education, annual income, ever-smokers, homebound status, baseline major chronic diseases, physical frailty, functional status, and cognitive impairment. HR, hazard ratio; CI, confidential interval; BMI, body mass index; WC, waist circumference; WWI, weight-adjusted waist index; WHtR, waist-to-height ratio; WHT.5R, waist-to-height^0.5. *$P < 0.05$

Supplementary Table S2. Sex-stratified sensitivity analysis of the assocation between five annual anthropometric dynamics and all-cause mortality (N=5245).

Anthropometric Metrics	Male			Female		
	Event / No. at risk	Adjusted HR (95% CI)	*P* value	Event / No. at risk	Adjusted HR (95% CI)	*P* value
**BMI change**						
Stable (within 2% change)	560 / 1566	1.00 (Ref)	-	625 / 1947	1.00 (Ref)	-
5% ≥ Decreased >2%	184 / 333	2.37 (1.86, 3.03)	<0.001*	266 / 541	2.02 (1.61, 2.53)	<0.001*
Decreased >5%	71 / 149	4.72 (3.32, 6.70)	<0.001*	134 / 218	6.10 (4.63, 8.03)	<0.001*
5% ≥ Increased >2%	56 / 135	3.83 (2.71, 5.41)	<0.001*	76 / 186	2.28 (1.42, 3.68)	0.001*
Increased >5%	19 / 61	3.38 (1.90, 6.02)	<0.001*	50 / 109	3.93 (1.99, 7.79)	<0.001*
**WC change**						
Stable (within 2% change)	653 / 1623	1.00 (Ref)	-	729 / 2067	1.00 (Ref)	-
5% ≥ Decreased >2%	113 / 252	2.81 (2.11, 3.73)	<0.001*	185 / 347	2.74 (2.17, 3.47)	<0.001*
Decreased >5%	31 / 89	6.00 (3.33, 10.80)	<0.001*	71 / 178	7.95 (5.80, 10.89)	<0.001*
5% ≥ Increased >2%	62 / 197	3.34 (2.38, 4.69)	<0.001*	119 / 268	2.96 (2.15, 4.07)	<0.001*
Increased >5%	31 / 83	8.20 (5.13, 13.11)	<0.001*	47 / 141	5.81 (3.59, 9.40)	<0.001*
**WWI change**						
Stable (within 2% change)	691 / 1737	1.00 (Ref)	-	795 / 2185	1.00 (Ref)	-
5% ≥ Decreased >2%	69 / 162	4.09 (2.76, 6.07)	<0.001*	101 / 216	4.07 (3.05, 5.41)	<0.001*
Decreased >5%	22 / 65	5.83 (3.11, 10.92)	<0.001*	47 / 135	6.71 (4.16, 10.81)	<0.001*
5% ≥ Increased >2%	75 / 183	3.88 (2.82, 5.34)	<0.001*	142 / 292	3.28 (2.40, 4.48)	<0.001*
Increased >5%	33 / 97	8.49 (4.99, 14.45)	<0.001*	66 / 173	3.38 (2.29, 4.98)	<0.001*
**WHtR change**						

Stable (within 2% change)	641 / 1632	1.00 (Ref)	-	718 / 2036	1.00 (Ref)	-
5% ≥ Decreased >2%	108 / 229	2.63 (2.02, 3.44)	<0.001*	163 / 323	2.53 (1.93, 3.33)	<0.001*
Decreased >5%	33 / 94	6.07 (3.33, 11.10)	<0.001*	80 / 181	5.47 (3.69, 8.12)	<0.001*
5% ≥ Increased >2%	72 / 196	3.34 (2.38, 4.70)	<0.001*	134 / 297	2.43 (1.82, 3.23)	<0.001*
Increased >5%	36 / 93	7.88 (5.12, 12.13)	<0.001*	56 / 164	4.83 (3.01, 7.74)	<0.001*
**WHT.5R change**						
Stable (within 2% change)	648 / 1632	1.00 (Ref)	-	722 / 2058	1.00 (Ref)	-
5% ≥ Decreased >2%	109 / 238	2.54 (1.92, 3.36)	<0.001*	169 / 330	2.53 (1.96, 3.25)	<0.001*
Decreased >5%	34 / 92	5.87 (3.29, 10.48)	<0.001*	81 / 182	5.48 (3.63, 8.28)	<0.001*
5% ≥ Increased >2%	65 / 195	3.02 (2.15, 4.24)	<0.001*	120 / 268	2.43 (1.82, 3.23)	<0.001*
Increased >5%	34 / 87	8.06 (5.23, 12.45)	<0.001*	59 / 163	4.71 (3.03, 7.30)	<0.001*

Note: the sex specific associations between anthropometric changes and all-cause mortality examined via multiple analysis and models adjusted for baseline age, race, education, annual income, ever-smokers, homebound status, major chronic disease status, physical frailty, functional status, and cognitive impairment. HR, hazard ratio; CI, confidential interval; BMI, body mass index; WC, waist circumference; WWI, weight-adjusted waist index; WHtR, waist-to-height ratio; WHT.5R, waist-to-height^0.5. *$P < 0.05$.

Supplementary Table S3. Sensitivity analysis of five anthropometry-mortality relationships using annual changes derived from two-point (first and last) observations (N=5245).

Anthropometric Metrics	Event / No. at risk	Adjusted HR (95% CI)	*P* value	Harrell C statistic (95% CI)
**BMI change**				
Stable (within 2% change)	1191 / 3522	1.00 (Ref)	-	0.751 (0.738, 0.763)
5% ≥ Decreased >2%	459 / 867	2.49 (2.10, 2.95)	<0.001*	
10% ≥ Decreased >5%	134 / 254	5.71 (4.50, 7.25)	<0.001*	
Decreased >10%	46 / 81	8.14 (4.95, 13.39)	<0.001*	
5% ≥ Increased >2%	138 / 340	2.65 (1.96, 3.56)	<0.001*	
10% ≥ Increased >5%	46 / 123	3.67 (2.25, 6.00)	<0.001*	
Increased >10%	27 / 58	7.99 (4.18, 15.29)	<0.001*	
**WC change**				
Stable (within 2% change)	1191 / 3522	1.00 (Ref)	-	0.749 (0.736, 0.762)
5% ≥ Decreased >2%	286 / 590	2.68 (2.27, 3.17)	<0.001*	
10% ≥ Decreased >5%	80 / 197	3.97 (2.33, 6.78)	<0.001*	
Decreased >10%	41 / 110	2.54 (1.05, 6.18)	0.04*	
5% ≥ Increased >2%	202 / 500	2.30 (1.75, 3.03)	<0.001*	
10% ≥ Increased >5%	65 / 177	6.59 (4.48, 9.68)	<0.001*	
Increased >10%	16 / 62	4.77 (2.63, 8.64)	<0.001*	
**WWI change**				
Stable (within 2% change)	1191 / 3522	1.00 (Ref)	-	0.747 (0.734, 0.760)
5% ≥ Decreased >2%	176 / 398	3.17 (2.55, 3.95)	<0.001*	
10% ≥ Decreased >5%	48 / 143	3.21 (1.81, 5.70)	<0.001*	
Decreased >10%	56 / 124	2.03 (1.22, 3.36)	0.007*	
5% ≥ Increased >2%	253 / 546	2.75 (2.28, 3.31)	<0.001*	
10% ≥ Increased >5%	79 / 197	3.53 (2.44, 5.11)	<0.001*	
Increased >10%	29 / 92	4.56 (2.79, 7.48)	<0.001*	
**WHtR change**				
Stable (within 2% change)	1191 / 3522	1.00 (Ref)	-	0.747 (0.733, 0.760)
5% ≥ Decreased >2%	274 / 565	2.68 (2.22, 3.22)	<0.001*	
10% ≥ Decreased >5%	81 / 195	3.75 (2.37, 5.92)	<0.001*	
Decreased >10%	63 / 137	3.08 (1.70, 5.57)	<0.001*	

5% ≥ Increased >2%	225 / 536	2.29 (1.81, 2.90)	<0.001*	
10% ≥ Increased >5%	71 / 194	4.97 (3.56, 6.94)	<0.001*	
Increased >10%	29 / 82	4.59 (2.70, 7.82)	<0.001*	
**WHT.5R change**				
Stable (within 2% change)	1191 / 3522	1.00 (Ref)	-	
5% ≥ Decreased >2%	276 / 573	2.68 (2.24, 3.20)	<0.001*	
10% ≥ Decreased >5%	84 / 194	3.82 (2.44, 5.99)	<0.001*	
Decreased >10%	61 / 135	2.98 (1.61, 5.50)	<0.001*	0.748 (0.735, 0.761)
5% ≥ Increased >2%	206 / 503	2.20 (1.72, 2.81)	<0.001*	
10% ≥ Increased >5%	72 / 190	6.24 (4.61, 8.44)	<0.001*	
Increased >10%	25 / 74	3.59 (2.14, 6.02)	<0.001*	

Note: the associations between anthropometric indices and all-cause mortality were examined via multiple analysis, the models adjusted for baseline age, sex, race, education, annual income, ever smokers, homebound status, major chronic disease status, physical frailty, functional status, and cognitive impairment. The annual percentage change is calculated = [100*(end measure - baseline measure)/baseline measure]/follow-up duration. HR, hazard ratio; CI, confidential interval; BMI, body mass index; WC, waist circumference; WWI, weight-adjusted waist index; WHtR, waist-to-height ratio; WHT.5R, waist-to-height^0.5. *$P < 0.05$.

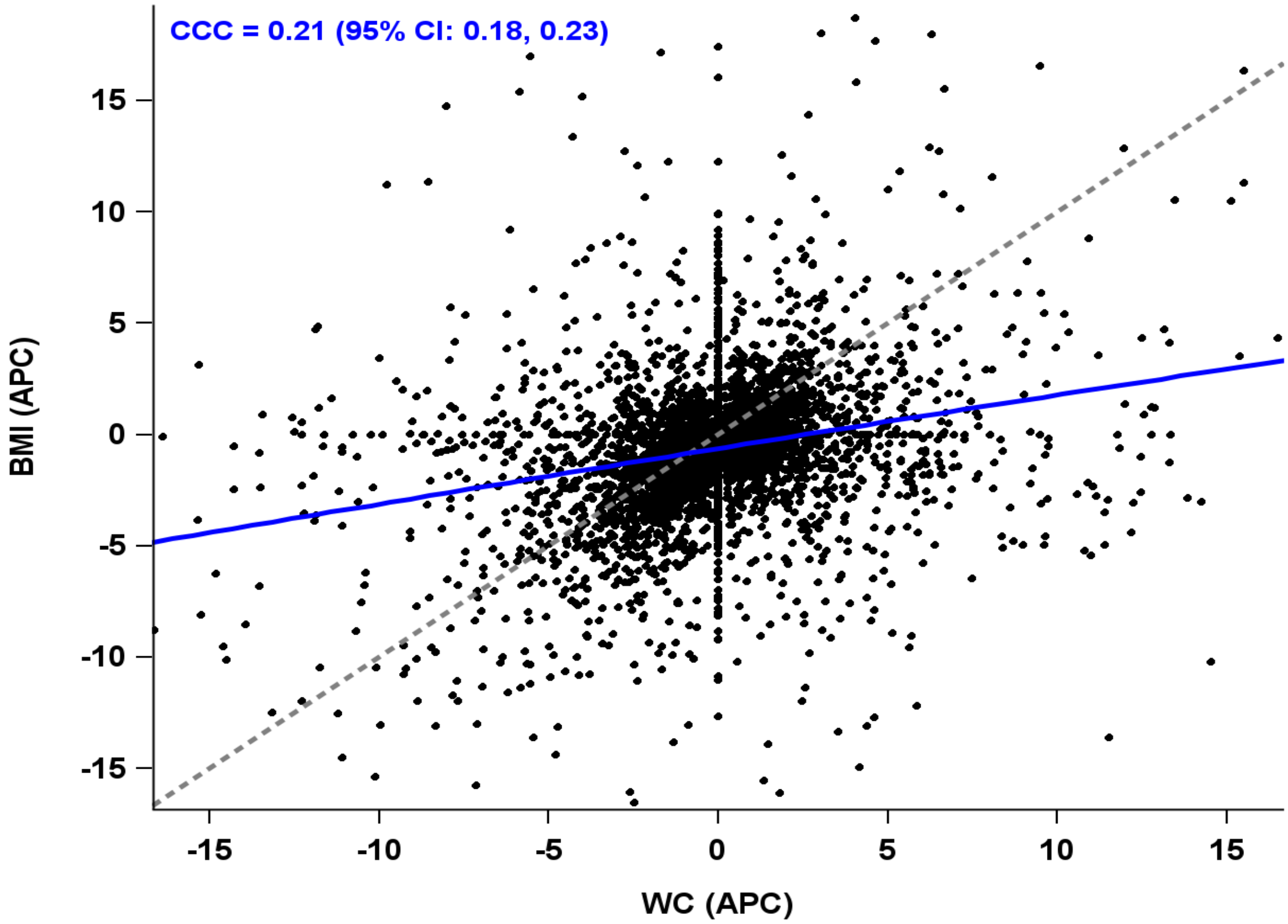

Supplementary Figure S1. Agreement plot and CCC for APC in BMI and WC. CCC, Concordance correlation coefficient; BMI, body mass index; WC, waist circumference; APC, annualized percentage change.

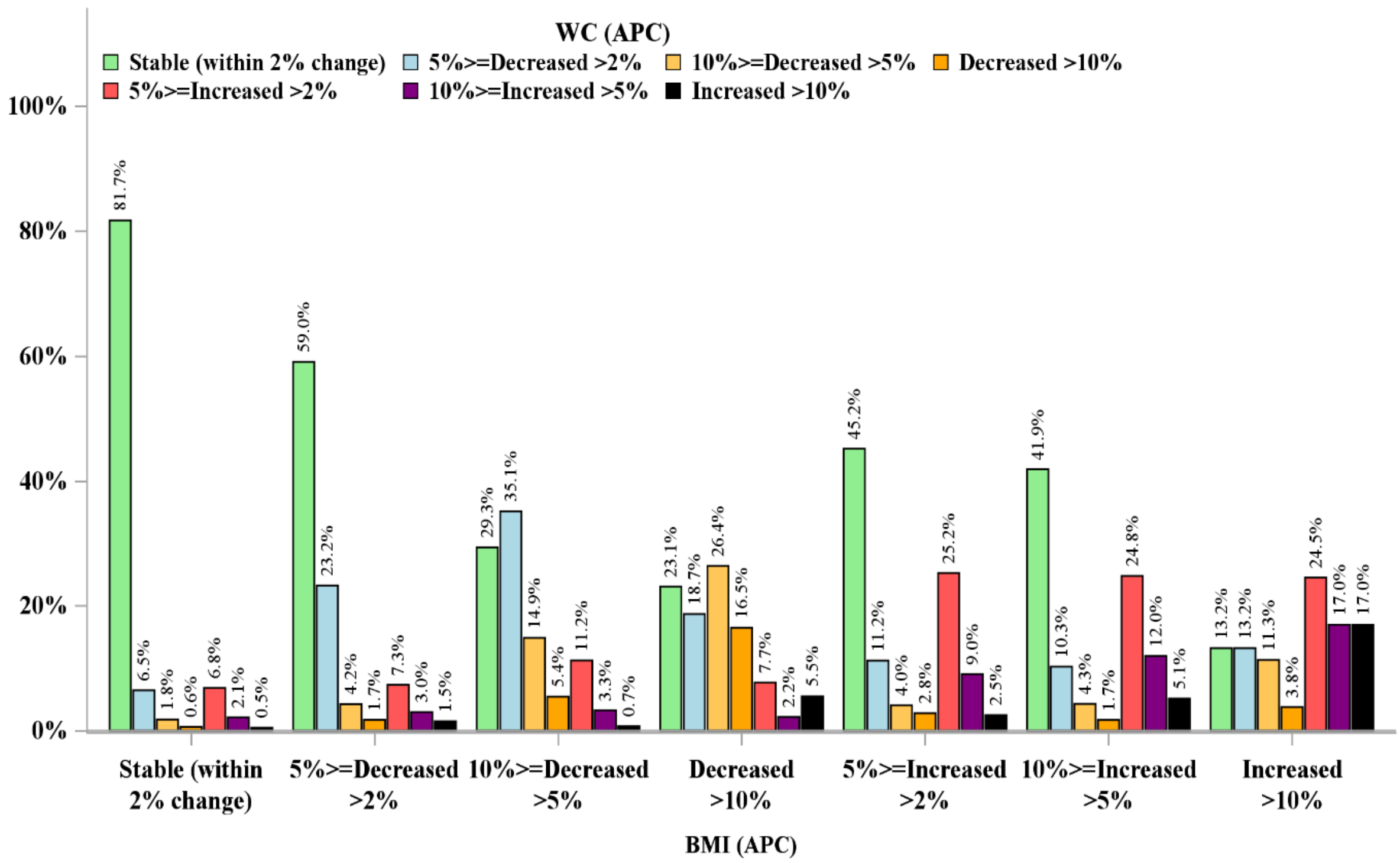

Supplementary Figure S2. Percentage distribution of annual WC change categories within BMI change categories. WC, waist circumference; BMI, body mass index; APC, annual percentage change.
